# Psychoactive Substances Taken with Mephedrone and HCV Infection

**DOI:** 10.3390/jcm10153218

**Published:** 2021-07-21

**Authors:** Michal Ordak, Tadeusz Nasierowski, Elzbieta Muszynska, Magdalena Bujalska-Zadrozny

**Affiliations:** 1 Department of Pharmacodynamics, Centre for Preclinical, Research and Technology (CePT), Medical University of Warsaw, 02-091 Warsaw, Poland; mbujalska@gmail.com; 2 Department of Psychiatry, Medical University of Warsaw, 02-091 Warsaw, Poland; tadeusz@wum.edu.pl; 3 Department of Medical Biology, Medical University of Bialystok, 15-089 Bialystok, Poland; ela3@onet.eu

**Keywords:** benzodiazepines, mephedrone, HCV infection

## Abstract

Background: In recent years, the observed frequency of hospitalization of patients taking mephedrone with other psychoactive substances has increased. There are no data in the literature on the effect of mephedrone use on liver function in patients, including the frequency of HCV infection. We have analysed the impact of taking mephedrone together with other psychoactive substances on the incidence of HCV infection. We have also analysed the effect of taking mephedrone with heroin, alcohol, and benzodiazepines on liver enzyme levels. Methods: The study included patients taking mephedrone with: alcohol (*n* = 115), heroin (*n* = 85) and benzodiazepines (*n* = 130) hospitalized in 2010–2018. The control group consisted of patients addicted to alcohol (*n* = 180), heroin (*n* = 221) and benzodiazepines (*n* = 152). Clinical data and laboratory findings were collected from medical records. Results: Taking mephedrone together with benzodiazepines is a statistically significant predictor of HCV infection in this group of patients, OR (8.44); 95% CI 5.63–12.64; *p* < 0.001). A statistically significant interaction of the group with HCV infection was observed, i.e., for the level of alanine transaminase (*p* < 0.001) and aspartate transaminase (*p* < 0.001). Increased levels of liver enzymes in each of the studied groups was characteristic in patients with HCV infection (*p* < 0.001). Taking additional mephedrone by this group of patients did not increase the level of liver enzymes. Conclusion: HCV infection is a statistically significant factor affecting the increase in liver enzymes levels in the group of patients taking mephedrone.

## 1. Introduction

In recent years, we have seen a rapid increase in the frequency of hospitalizations of patients who abuse new psychoactive substances [1]. Based on data from the 2014 European Drug Report of the European Monitoring Centre for Drugs and Drug Addiction (EMCDDA), a record number of 101 new psychoactive substances were identified in Europe [2]. In 2017, the EMCDDA reported a decrease in the number of such drugs [3]. Unfortunately, notwithstanding, the number of hospitalizations of individuals who abuse new recreational drugs is still very high. Introduction of various solutions by the European Union has not mitigated the problem [1,4].

Mephedrone is one of the most common psychoactive substances whose intake has led to a higher frequency of patient hospitalization in recent years [5,6]. On 3 August 2010, the Council of the European Union decided to subject mephedrone to control measures and impose criminal penalties as provided for under national legislation in the European Union [7]. This has not reduced the problem of mixing mephedrone with psychoactive substances. An article published in *Lancet* in 2016 indicates that in many countries, such as the United Kingdom, a growing number of mephedrone-associated fatalities has also been observed. According to the authors, the number of fatalities involving mephedrone rose from 1% in 2014 to 1.5% in 2015. This percentage is comparable to the deaths associated with the intake of methyl-enedioxy-methylamphetamine (MDMA) [5]. The strong addictive potential of mephedrone raises the risk of future hospitalizations of the same people [8]. According to the data published in 2018 in *Lancet Psychiatry*, the younger the person, the higher the dose of mephedrone they tended to take, and the longer the binge [6].

Mixing novel psychoactive substances with other drugs does not have a positive effect on liver function. Unfortunately, a search of the literature does not reveal any research on liver enzymes carried out on a representative group of subjects, involving patients using new psychoactive substances, including mephedrone. Only singular studies on rats indicate that both mephedrone and other cathinones increase body temperature. On the second or third day of hyperthermia, there may also be signs of acute liver damage and, in particularly severe cases, of acute liver failure [8,9]. There is only one report on the mechanism of hepatotoxicity of mephedrone; however, this is not data based on long-term clinical research, but only on a study of HepG2 cell lines [10]. The literature also describes a case of acute liver failure after using synthetic cathinones [11]. In vitro research has demonstrated that mitochondrial dysfunction and oxidative stress contribute to liver damage associated with these chemical compounds [10,12].

One of the most common infections in the group of individuals taking various types of drugs, including new psychoactive substances, is caused by hepatitis C virus (HCV) [13]. According to US statistics, the spread of HCV in the population of active drug users ranges between 63% and 90% [14,15,16]. A consequence of HCV infection is damage to the liver parenchyma, which further reduces the chance of achieving a therapeutic effect. HCV infection causes damage to the liver parenchyma, and new psychoactive substances may have hepatotoxic effects. Both these factors may contribute to the lack of a therapeutic effect in the group of people taking new psychoactive substances. This may be caused by the fact that many psychotropic drugs are metabolized in the liver. A single publication regarding HCV infection in a group of patients who use new psychoactive substances indicates that from 2011 to 2014, HCV infections doubled as a result of the use of NPSs. As for the group of young people, the percentage of hepatitis C rose by a factor of seven. In the group of people injecting legal highs, the prevalence of HCV doubled from 37% to 74% (2011–2014). In younger people (aged <25 years) there was a seven-fold increase in the frequency of HCV infections, that is, from 12% to 76%. In the group of people taking legal highs for less than two years, this percentage increased from 13 to 42. This is because 79% of those taking new psychoactive substances did not use a condom during their last intercourse, 65% were in prison and 57% were homeless in the previous year [17].

The data on HCV infection published in the literature to date relate to patients who abuse psychoactive substances such as alcohol, heroin or cocaine. There is a strong correlation between HCV infection and alcoholic liver disease. Patients who abuse alcohol often present with changes in the immune system that predispose them to HCV infection. In those patients, HCV infection correlates with greater organ damage [18]. The risk of HCV infection is also high in users of injectable heroin. According to Zhou et al., HCV-infected heroin users had significantly higher viral loads compared to HCV-infected non-heroin users [19]. The above also applies to cocaine. The multivariate analysis conducted by Oliveira-Filho et al. has shown a relationship between HCV infection and tattoos, shared use of paraphernalia, daily cocaine use and a long history of cocaine use [20]. In an article published in 2016 in the American Journal of Public Health, the authors indicate that the use of benzodiazepines is associated with an increased percentage of HCV seroconversion [21]. There is no data in the literature on the effects of the consumption of NPSs (including mephedrone) with other psychoactive substances on the incidence of HCV infection. Only a single publication, namely the one from 2019, indicates that there is an increased risk of HCV infection in the group of patients injecting new psychoactive substances. However, this type of data is general and does not include tests focused on individual drugs taken with new psychoactive substances, i.e., their effects on the risk of HCV infection [22].

A very important argument supporting the need for this type of research is the crucial importance of liver function in the therapy of patients who take NPSs. In recently published studies, the authors have pointed out that the decrease in the frequency of hospitalization in patients taking liver regenerative substances while reducing the amount of co-administered psychotropic drugs is statistically significant [23]. This prompted us to distinguish three research objectives in the group of patients who binged on mephedrone between 2010 and 2018.

Literature data indicate that patients combine new psychoactive substances with other drugs which unfortunately also blurs the clinical picture. It happens that, in fact, people declaring taking mephedrone continuously take a product in which the amount of mephedrone is negligible and is mostly found in various toxic admixtures [24,25,26]. Exemplary research by Van Hout et al. indicate that patients taking new psychoactive substances combine them with substances that have a sedative and relaxing effect [27].

Because people taking mephedrone combine it with other psychoactive substances, the first goal was to see if any of these combinations increased the risk of HCV infection.

The second goal was to check whether there are statistically significant differences in the level of liver enzymes between the group of people taking mephedrone with alcohol, heroin, or benzodiazepines, and specifically with the group of people taking this type of substance, but without mephedrone. In other words, the comparative group consisted of patients addicted to alcohol, heroin, or benzodiazepines alone.

The third goal was to check whether there is a statistically significant interaction in the group of people with HCV infection, i.e., the effect on the level of the liver enzymes tested, namely alanine transaminase (ALT) and aspartate transaminase (AST).

## 2. Methods

### 2.1. Study Design and Participants

The study included 330 patients taking mephedrone with other psychoactive substances in 2010–2018 (for 2 days or more). All subjects in our sample binged on mephedrone in addition to one other psychoactive substance: alcohol, heroin and benzodiazepines. Regarding individual substances, heroin was injected together with mephedrone, while the remaining substances were taken with mephedrone intranasally. The study included patients who were hospitalised in the psychiatric hospital because they took mephedrone regularly, that is, for a minimum of two days. Patients were recruited by psychiatrists working at the Nowowiejski Psychiatric Hospital in Warsaw. The criterion for exclusion was concomitant mental disorders. Patients with comorbid mental illnesses such as schizophrenia were excluded from the study. They did not have comorbidities. Patients with chronic diseases, including autoimmune, cancer, and cardiovascular diseases, were also excluded. The length of stay of the examined patients in the psychiatric hospital during each hospitalization was 14 days. Patients with diseases that could affect the results of hepatic parameters were also excluded from the study. An example of such disease is liver cirrhosis. This made us examine how taking particular psychoactive substances together with mephedrone affects the level of liver enzymes during the patient’s stay in the hospital. None of the subjects took mephedrone alone, as noted in the limitations of this study. The comparison group consisted of 221 heroin addicted patients, 180 alcohol addicts and 152 benzodiazepines addicts. Therefore, it was possible to relate the group of people taking mephedrone with individual psychoactive substances, namely to the groups of patients addicted only to alcohol, heroin, and benzodiazepines. To put it in another way, the comparison concerned those not taking mephedrone, but addicted to alcohol, heroin, or benzodiazepines alone.

### 2.2. Biochemical Assessment

The presence of mephedrone and other psychoactive substances was confirmed analytically with urine tests done by My Lab, based on a qualitative study. In the compared groups of patients, the levels of alanine transaminase (ALT) and aspartate transaminase (AST) were measured immediately after the patient was admitted to the hospital. The measurement was performed by ALAB laboratory staff. Anti-HCV antibodies were measured in the six compared groups of patients, namely those addicted to alcohol, heroin, or benzodiazepines, as well as in groups taking mephedrone alongside these psychoactive substances. Genetic material (RNA) of HCV was detected to confirm the current infection. The study of the presence of HCV RNA, preceded by reverse transcription, was performed using Real Time-PCR. HCV infection was first detected in a patient during his or her hospitalization. The variables collected included the following data: substances taken with mephedrone, sex, age and HCV infection.

### 2.3. Ethics

This was a retrospective study of hospitalised patients. No off standard proceedings were used. This was the usual procedure for admitting patients to a psychiatric hospital. Therefore, the study did not require the consent of the Bioethics Committee. The director of the Nowowiejski Psychiatric Hospital agreed to conduct the study. In addition, the authors of the study received materials on the safety policy of the Nowowiejski Hospital. After becoming acquainted with this type of material, the Data Protection Inspector of the Independent Provincial Group of Public Psychiatric Healthcare Institutions in Warsaw provided additional training for this purpose, which finally allowed the study to take place. The consent obtained, preceded by a specialist training course, allowed the authors of the article to use the collected data for scientific purposes only.

### 2.4. Statistical Analyses

Statistical analysis was conducted using the IBM SPSS Statistics 25 Package. The chi-squared test was used to check whether there is a statistically significant relationship between the nominal variables, e.g., between a group of people and the incidence of HCV infection. To determine the level of this relationship, the Cramér’s V contingency coefficient was used. Two-way ANOVA was used to examine a group of people from the viewpoint of statistically significant influence on the level of liver enzymes, as well as its interaction with HCV infection. Simple main effects analysis was used to examine in detail a statistically significant interaction in the analysis of variance. Logistic regression analysis made it possible to prove whether the risk of HCV infection in the studied group could be predicted. The statistically significant level was *p* < 0.05.

## 3. Results

### 3.1. Characteristics of the Studied Patient Population

Out of 330 patients taking mephedrone, 34.8% (*n* = 115) combined the drug with alcohol alone, 25.8% (*n* = 85) with heroin and 39.4% (*n* = 130) with benzodiazepines. The first table presents sociodemographic data on the studied groups. There was a statistically significant relationship between the studied group and their gender, χ^2^(5) = 475.49; *p* < 0.001. In the group of patients addicted to benzodiazepines, the largest proportion were women. The opposite situation was typical for other groups of people, i.e., those taking mephedrone with certain substances, as well as addicted to alcohol or heroin. A statistically significant majority of the surveyed people were city dwellers (*p* < 0.001). There was also a statistically significant correlation between age and group, χ^2^(20) = 373.29; *p* < 0.001. In the group of patients addicted to benzodiazepines, most were aged from 46 to 55 years, while in the remaining groups the age range was from 36 to 45 years (Table 1).

### 3.2. Taking Mephedrone with Psychoactive Substances and Frequency of HCV Infection

The table below (Table 2) presents data on the frequency of HCV infection in the compared groups of patients at the time of admission to the hospital. This was the first time HCV infection was detected. There was a statistically significant relationship between group and HCV infection, V = 0.56; *p* < 0.001 (Cramér’s phi coefficient). In the group of 172 people addicted to alcohol, as well as those taking mephedrone with alcohol, a statistically significant majority did not develop HCV (*p* < 0.001). The same is valid for heroin addicts (*p* < 0.001). In contrast, in the group of patients taking mephedrone plus heroin compared to patients addicted to heroin alone, there was a slightly higher percentage of those infected with HCV. Benzodiazepines were particularly highlighted. In the group of benzodiazepine addicts, none of the patients had HCV infection. The opposite is characteristic of patients combining mephedrone with this group of substances. HCV infection was observed in a statistically significant majority. Taking mephedrone with benzodiazepines is a statistically significant predictor of HCV infection in this group of patients, OR (8.44); 95% CI 5.63–12.64; *p* < 0.001). It is also noteworthy that in the group of patients taking mephedrone with individual psychoactive substances, a greater percentage was infected with HCV compared to patients taking this type of substance, i.e., without mephedrone. Both sex and age of patients in particular groups did not have a statistically significant influence on the frequency of HCV infection (*p* > 0.05).

### 3.3. Liver Enzyme Levels

Table 3 presents descriptive statistics on the levels of liver enzymes, i.e., alanine and aspartate transaminases, in the compared groups of patients (Table 3). There were statistically significant differences between individual groups in terms of the levels of liver enzymes observed. In patients addicted to heroin, the levels of alanine transaminase were statistically significantly lower compared to patients taking mephedrone plus heroin (*p* = 0.02). The same applies to the comparison of benzodiazepine-dependent patients with patients who combine this group of substances with mephedrone. (*p* = 0.03). In the case of alcohol, no such statistically significant differences were observed (*p* = 0.8). Similar results were characteristic for aspartate transaminase: heroin vs. mephedrone plus heroin (*p* = 0.01), benzodiazepines vs. mephedrone plus benzodiazepines (*p* = 0.002), alcohol vs. mephedrone plus alcohol (*p* = 0.34).

What is noteworthy is the statistically significant interaction of the group of patients with HCV infection. A simple main effect analysis was used to investigate the interactions in detail. Therefore, it was possible to examine the differences in groups of patients additionally divided according to whether they were infected with HCV or not. The obtained results indicated that in the group of patients without HCV infection, there were no statistically significant differences between individual groups of patients in terms of liver enzyme levels (*p* > 0.05).

The analysis of simple main effects also showed that in each of the six studied groups of patients there were statistically significant differences between patients infected or not infected with HCV. In individual groups of patients, i.e., infected with HCV, the levels of both enzymes turned out to be statistically significantly higher compared to similar groups of patients without HCV infection (*p* < 0.001).

The opposite situation was typical of HCV infected patients. In those patients, greater differences were observed than in groups of patients without any consideration for the HCV infection. In heroin addicts, and those co-infected with HCV, the levels of alanine transaminase were statistically significantly higher compared to patients also infected with HCV taking mephedrone plus heroin (*p* = 0.009). Similar differences also applied to HCV infected patients, i.e., comparisons of alcohol vs. mephedrone plus alcohol (*p* = 0.03). It is noteworthy that when HCV infection was not taken into consideration, there were no statistically significant differences between patients addicted to alcohol and those who combined alcohol with mephedrone. Similar differences were observed for aspartate transaminase in HCV infected patients: heroin vs. mephedrone plus heroin (*p* < 0.001), alcohol vs. mephedrone plus alcohol (*p* < 0.001). Gender, as well as age, in particular groups did not have a statistically significant effect on liver enzyme levels (*p* > 0.05).

## 4. Discussion

Our study was aimed, firstly, at examining the prevalence of HCV infection in a group of patients taking mephedrone with other psychoactive substances, as well as liver enzymes. Conducting this research has allowed us to broaden our knowledge about the problem of users of mephedrone with other psychoactive substances that has been growing for many years. This substance has a strong addictive potential, which further warrants carrying out this type of research. Another argument that prompted this study was the many fatalities associated with mephedrone abuse, such as in London [5]. In view of the fact that patients do not take mephedrone alone, the study included patients addicted solely to psychoactive substances, i.e., substances combined with mephedrone. Of all the psychoactive substances, only benzodiazepines showed a statistically significant relationship with the occurrence of HCV infection. None of the patients addicted to benzodiazepines, i.e., not taking additional mephedrone, were infected with HCV.

The EMCDDA country prevalence for HCV infection indicates a prevalence of around 60% HepC+ in Poland at a national level [28]. However, there are conflicting literature data, i.e., including date in which the authors found that this percentage was lower. In the sample studies published by Roux et al., the authors indicated that the frequency of HCV infection in the group of patients addicted to heroin was 35% [29]. In our study, this percentage turned out to be even lower. This may be due to certain factors. Firstly, the risk of HCV infection is higher in patients combining heroin with alcohol and cocaine [29,30,31], and in our study we studied patients addicted to heroin only. The effects of additional alcohol intake, such as disinhibition, loss of judgment, improved mood or depression may contribute to the involvement of patients in risky sexual behaviour and, consequently, there is an increased likelihood of HCV transmission. Secondly, future studies should be based on a more representative group of patients, which would allow for a more detailed analysis of risk factors and HCV infection.

There is little data in the literature suggesting that benzodiazepine addiction may heighten the risk of HCV infection. Research conducted in recent years indicates that benzodiazepine abuse is strongly correlated with an increase in risk-taking behaviour, such as unprotected sex or needle sharing [32]. For the first time, the relationship between benzodiazepine intake and HCV infection was described in the retrospective PWID (persons who inject drugs) cohort study. The authors found a correlation between HCV seropositivity and the use of benzodiazepines [33]. Other studies, namely a cohort analysis conducted in the United States, also observed a relationship between benzodiazepine intake and HCV infection [34]. Recent research, i.e., carried out by Bach et al., confirmed a higher incidence of HCV infection among patients taking benzodiazepines, and showed that BZD use remained independently associated with a higher rate of HCV seroconversion [21]. Our results indicate that 61.5% of patients who combine mephedrone with benzodiazepines have HCV infection. This may be connected with the fact that both the use of mephedrone and benzodiazepines significantly impair cognitive function. In their study, Freemen et al. showed that cognitive functions significantly deteriorated in people taking mephedrone compared to the control group [35]. The same applied to patients taking benzodiazepines. A meta-analysis carried out in 2020 indicated that overuse of benzodiazepines can significantly affect cognitive functions [36]. Cognitive deficits aggravate the difficulty in achieving lasting abstinence [37]. Disturbances of consciousness resulting from the influence of psychoactive substances, aggression, or loss of control over one’s body may expose one to experience unwanted or risky sexual interaction. This may increase the risk of HCV infection. In view of the above, it seems necessary to develop a strategy to prevent benzodiazepine addiction, including patients on mephedrone binge. This could reduce the percentage of patients with HCV infection, and thus the associated morbidity and mortality. In their study, Arends et. al. found that different types of impulsivity predicted the risk of HCV, HIV and HBV infections [38]. For example, motor impulsivity makes it possible to predict risky behaviour related to alcohol consumption. Another study found that in HCV-infected patients, all domains of impulsivity were higher compared to those without the viral disease [39].

It would be useful to include research focused on the efficacy of targeting drug addiction in reducing health related problems. The level of knowledge about the risk of HCV infection should be raised among drug users. Interventions to reduce HCV infection should take place in the street and in nightclubs, i.e., in places attended by people taking mephedrone, among other substances. Hagan et al. have reviewed studies evaluating interventions and emphasized that the probability of success increases when preventive strategies are combined. Interventions containing many components, such as reducing or eliminating drug injections, providing sterile injection and drug preparation equipment, and counselling on how to behave in a less risky way have proved to be more effective [40]. In recent years, significant changes have been made in the treatment of hepatitis C related to the introduction of the widespread use of interferon-free therapies, consisting in the administration of drugs with direct antiviral activity, affecting specific HCV proteins (DAA, direct-acting antivirals). Treatment of hepatitis C with DAA is currently recommended by the World Health Organization (WHO). The goal of the EMCDDA and WHO is to eliminate HCV infection by 2030. Future research should also include DAA therapy in patients taking new psychoactive substances with other substances. The literature data indicate that the favourable safety profile of DAA allows for the qualification for treatment of patients from groups previously defined as difficult to treat due to the significant advancement of liver disease, coexistence of chronic kidney disease, and HIV co-infection. [41,42].

Our preliminary analyses of liver enzyme levels have shown that taking mephedrone with heroin or benzodiazepines increases those levels. However, a statistically significant interaction of the group of patients with HCV infection was observed later. This allowed determination of whether mephedrone was responsible for the increased level of liver enzymes or HCV infection. in the compared groups of patients. It turned out that statistically significantly higher levels of liver enzymes were characteristic of all groups of patients who are additionally infected with HCV. The groups of patients without concomitant HCV infection do not differ in terms of the level of liver enzymes. In addition, in the group of patients addicted to heroin or benzodiazepines and simultaneously with HCV infection, the liver enzyme levels were statistically significantly higher compared to the group of patients combining these substances with mephedrone and with HCV infection. This indicates that it is not mephedrone but HCV infection that is a statistically significant factor, i.e., it influences the increase of liver enzyme levels.

Hepatitis C is a valid substance-related medical issue, as well as a major diagnostic challenge due to its mildly symptomatic course and serious consequences of long-term damage to the liver by the pathogen. The uncharacteristic, often even silent clinical course, and thus late diagnosis, cause the virus to gradually destroy hepatocytes, eventually leading to cirrhosis, the serious complications of which are often the first symptom of inflammation that has been going on for many years. A typical feature of HCV infections is damage to the liver parenchyma, which may lead to liver failure or cirrhosis. Active inflammation contributes to progressive destruction of the normal liver parenchyma, which may eventually develop into functional liver failure. As a result of taking psychoactive substances and simultaneous occurrence of HCV infection, in the groups of patients we studied the parenchymal cells of the liver were damaged, thus the release of transaminases from the liver into the blood may have occurred, increasing their concentration. [43,44].

The results obtained are confirmed by the literature data showing that HCV infection in the group of patients addicted to alcohol increases the level of liver enzymes to a statistically significant level. [45]. Studies conducted in a group of 819 alcohol-dependent patients with co-infection with HCV also seem to indicate the same [46]. Similar data are characteristic for heroin addicted patients. In one of the published articles, the authors pointed to the fact that in the group of heroin-addicted patients, elevated levels of liver enzymes occurred in patients with HCV infection. [47]. Similar data have been reported, for example, for pregnant patients treated with methadone or buprenorphine. In the group of pregnant patients infected with HCV, regardless of the duration of treatment, the levels of liver enzymes were higher than in those without HCV infection [48]. In the group of injecting drug users with HCV infection, an increased risk of subsequent hospitalizations has been observed [16]. For this reason, as far as possible, education in the medical community should be conducted to minimize the number of HCV infections in the group of patients taking psychoactive substances.

## 5. Conclusions

HCV infection is a statistically significant factor affecting the increase in liver enzymes levels in the group of patients taking mephedrone with other psychoactive substances.

## 6. Limitations

This is a quantitative study, but it lacks data on the determination of mephedrone. The reason for this is that quantitative methods for the determination of new psychoactive substances have only been developed in recent years, and the problem of hospitalization of patients who abuse such substances arose much earlier. Another limitation of this study is the fact that patients do not take mephedrone alone but combine it with other psychoactive substances. Therefore, it is difficult to determine the clinical impact of mephedrone alone, including on the liver function. For this reason, comparative groups were included in this study, i.e., patients addicted only to alcohol, heroin, or benzodiazepines. Another limitation is the lack of quantification of anti-HCV antibodies. The conducted studies are the first on the level of liver enzymes in patients taking a new psychoactive substance; however, further studies are needed where, in addition, more specific biochemical markers of the liver function will be tested, as well as supplementation with preparations regenerating this organ.

## Figures and Tables

**Table 1:** Sociodemographic data of the compared groups.

Variable		Patients Taking Mephedrone *n* (%)			Patients Addicted to Alcohol *n* (%)	Patients Addicted to Heroin *n* (%)	Patients Addicted to Benzodiazepines *n* (%)
		With Alcohol	With Heroin	With Benzodiazepines			
Sex	Male	109 (94, 8)	76 (89, 4)	121 (93, 1)	143 (79, 4)	199 (90)	41 (27)
	Female	6 (5, 2)	9 (10, 6)	9 (6, 9)	37 (20, 6)	22 (10)	111 (73)
Age	15–25	43 (37, 4)	12 (14, 1)	13 (10)	8 (4, 4)	19 (8, 6)	9 (5, 9)
	26–35	44 (48, 3)	45 (52, 9)	85 (65, 4)	51 (28, 3)	53 (24)	16 (10, 5)
	36–45	23 (20)	26 (30, 6)	28 (21, 5)	62 (34, 4)	118 (53, 4)	41 (27)
	46–55	4 (3, 5)	2 (2, 4)	3 (2, 3)	36 (20)	31 (14)	56 (36, 8)
	56–65	1 (0, 9)	0 (0)	1 (0, 8)	23 (12, 8)	0 (0)	30 (19, 7)
Place of residence	City	113 (98, 3)	84 (98, 8)	126 (96, 9)	172 (95, 5)	210 (95)	145 (95, 4)
	Village	2 (1, 7)	1 (1, 2)	4 (3, 1)	8 (4, 5)	11 (5)	11 (4, 6)

**Table 2:** The incidence of HCV infection in the compared groups.

Substance	HCV Infection		Statistical Test Results *
	−	+	
Mephedrone + alcohol	79 (68.7)	36 (31.3)	χ^2^(1) = 16.08; *p* < 0.001
Alcohol	172 (95.6)	8 (4.4)	χ^2^(1) = 149.42; *p* < 0.001
Mephedrone + heroin	34 (40)	51 (60)	χ^2^(1) = 3.4; *p* = 0.07
Heroin	196 (88.7)	25 (11.3)	χ^2^(1) = 132.31; *p* < 0.001
Mephedrone + benzodiazepines	50 (38.5)	80 (61.5)	χ^2^(1) = 6.92; *p* = 0.009
Benzodiazepines	152 (100)	0 (0)	-

* chi-square test.

**Table 3:** The levels of liver enzymes in the compared groups of patients, i.e., divided according to whether they are infected or not with HCV.

Group		Variable	HCV						Entire Group		
			−			+					
			M	Me	SE	M	Me	SE	M	Me	SE
Mephedrone + alcohol		ALT	29.65	22	2.98	57.24	46	7.64	38.58	26	3.42
		AST	31.88	21	4.31	65	44	9.35	42.6	25	4.46
Alcohol		ALT	37.28	27,5	2.63	116,75	87	24.31	40.81	29	2.98
		AST	42.84	31	3.6	192.25	151	51.44	49.48	32	4.66
Mephedrone + heroin		ALT	27.03	22	2.39	94.61	50	35.83	65.65	30	20.75
		AST	32	25	4.28	87.53	60	21.2	64.04	36	12.69
Heroin		ALT	31.87	25	1.73	139.84	103	28.79	44.08	27	4.23
		AST	32.39	27	1.51	139.68	107	22.9	44.52	28	3.68
Mephedrone + benzodiazepines		ALT	39.02	27	5.24	61.82	46.5	5.85	53.45	39	4.28
		AST	46.47	29	6.93	67.34	49.5	8.02	59.93	36	5.78
Benzodiazepines		ALT	30.2	21	3.8	-	-	-	30.2	21	3.8
		AST	32.55	27	3.39	-	-	-	32.55	27	3.39
Statistical significance	Differences between groups *	ALT	*p* < 0.001								
		AST	*p* < 0.001								
	Interaction of group and HCV infection *	ALT	*p* < 0.001								
		AST	*p* < 0.001								

* two-way ANOVA.

## Data Availability

The data are not publicly available due to its sensitive nature, to perspective the privacy of those enrolled in our study.
